# Supplementary material for: Monocentric observational cohort study to investigate the transmission of third-generation cephalosporin-resistant Enterobacterales in a neonatal intensive care unit in Heidelberg, Germany
Source: Microbiol Spectr. 2023 Sep 22;11(5):e02038-23. doi: 10.1128/spectrum.02038-23 (PMC10581168; doi:10.1128/spectrum.02038-23)

**Supplementary method**

**DNA extraction**

The isolates were regrown on BD™ Columbia Agar with 5% Sheep Blood (Becton Dickinson GmbH, Heidelberg, Germany) at 37 °C. DNA was extracted using the DNeasy Blood and Tissue Kit (Qiagen GmbH, Hilden, Germany) according to the manufacturer’s protocol.

**Library preparation and sequencing**

Libraries were prepare using the Nextera DNA Flex Library Prep Kit (Illumina). Each libraries were individually check on a Agilent 4200 TapeStation System (Agilent) and quantified using Quant-iT™ PicoGreen™ dsDNA Assay Kit (Thermo Fisher Scientific). Each librairies were sequenced on a MiSeq Illumina platform (short-read sequencing, 2 × 300 bp)

**Post sequencing procedure**

Raw sequences were controlled for quality using fastp (v0·23·2 with parameters -q = 30 and -l = 45) and assembled with SPAdes 3.15.5 (with the option —careful and—only-assembler)(32, 33). Draft genomes were curated by removing contigs with a length <500 bp and/or coverage <10×. The quality of the final draft was quality-controlled using Quast (v5.0.2) (34).

**Genomic analysis**

The complete draft genomes were processed through available databases using Abricate (<https://github.com/tseemann/abricate>) to identify antimicrobial resistance (NCBI, CARD, ARG-ANNOT, ResFinder, MEGARES databases) and plasmid type (PlasmidFinder database) to identify the Inc type of the plasmid (35, 36). The species identification of each draft genome was done using mash (sub-command screen) by screening each draft genome to a database composed of a representative genome of each species present in the Microbial Genomes resource (https://www.ncbi.nlm.nih.gov/genome/microbes/). Furthermore, each draft genome was aligned to its representative genome reference from the Microbial Genomes resource using SKA (using the script generate_ska_alignment.py from Gubbins 3.2.1 with default option). The alignment was then analysed with Gubbins 3.2.1 (using the script run_gubbins.py with default option) to define SNPs distance and phylogenetic relationship was determined using the R package samestrains following the methodology of Duvall et al. (37).

**Supplementary Table 1. Sequencing statistics**

| **isolate** | **accession** | **bioproject** | **%_identity** | **species** | **MLST** | **coverage** | **# contigs** | **Total length** | **GC (%)** | **N50** |
| --- | --- | --- | --- | --- | --- | --- | --- | --- | --- | --- |
| KE9619 | SAMN34137585 | PRJNA954276 | 0.990 | Citrobacter europaeus | - | 49 | 20 | 5424646 | 51.98 | 528329 |
| D2921 | SAMN34137593 | PRJNA954276 | 0.984 | Citrobacter freundii | 98 | 32 | 53 | 5269735 | 51.59 | 424577 |
| D2922 | SAMN34137587 | PRJNA954276 | 0.984 | Citrobacter freundii | 98 | 24 | 56 | 5267119 | 51.59 | 348494 |
| KE10208 | SAMN34137586 | PRJNA954276 | 0.985 | Citrobacter freundii | 64 | 68 | 65 | 5064808 | 51.75 | 213406 |
| KE10216 | SAMN34137588 | PRJNA954276 | 0.986 | Citrobacter freundii | 64 | 51 | 60 | 5222141 | 51.54 | 278885 |
| KE7375 | SAMN34137591 | PRJNA954276 | 0.985 | Citrobacter freundii | 64 | 54 | 60 | 5111112 | 51.56 | 177860 |
| KE8723 | SAMN34137592 | PRJNA954276 | 0.982 | Citrobacter freundii | 123-1LV | 39 | 50 | 5104335 | 52.04 | 465331 |
| KE9609 | SAMN34137589 | PRJNA954276 | 0.988 | Citrobacter freundii | 153 | 44 | 22 | 5092380 | 51.74 | 1105506 |
| KE9919 | SAMN34137590 | PRJNA954276 | 0.986 | Citrobacter freundii | 114 | 35 | 39 | 5150198 | 51.69 | 419418 |
| KE10064 | SAMN34137594 | PRJNA954276 | 0.966 | Citrobacter portucalensis | - | 64 | 17 | 5088138 | 51.66 | 837345 |
| KE9581 | SAMN34137595 | PRJNA954276 | 0.982 | Enterobacter asburiae | 657 | 58 | 15 | 4559040 | 56.07 | 860267 |
| KE9595 | SAMN34137596 | PRJNA954276 | 0.982 | Enterobacter asburiae | 657 | 65 | 15 | 4558621 | 56.07 | 860261 |
| KE9305 | SAMN34137597 | PRJNA954276 | 0.987 | Enterobacter bugandensis | 599 | 31 | 55 | 5147061 | 55.57 | 195441 |
| KE6465 | SAMN34137598 | PRJNA954276 | 0.990 | Enterobacter cloacae | 477 | 45 | 22 | 4784280 | 54.97 | 828815 |
| KE8543 | SAMN34137599 | PRJNA954276 | 0.959 | Enterobacter cloacae | 548 | 44 | 27 | 5204867 | 54.75 | 355440 |
| D1645 | SAMN34137607 | PRJNA954276 | 0.988 | Enterobacter hormaechei | 664 | 17 | 47 | 4887627 | 55.42 | 264593 |
| D1647 | SAMN34137626 | PRJNA954276 | 0.988 | Enterobacter hormaechei | 664 | 122 | 77 | 5042948 | 55.23 | 187486 |
| D1844 | SAMN34137646 | PRJNA954276 | 0.988 | Enterobacter hormaechei | 50 | 132 | 36 | 4782449 | 55.4 | 295696 |
| D1845 | SAMN34137609 | PRJNA954276 | 0.961 | Enterobacter hormaechei | 168 | 74 | 61 | 4776353 | 55.23 | 135628 |
| D1846 | SAMN34137610 | PRJNA954276 | 0.988 | Enterobacter hormaechei | 50 | 147 | 39 | 4747614 | 55.41 | 260034 |
| D1929 | SAMN34137628 | PRJNA954276 | 0.962 | Enterobacter hormaechei | 78 | 59 | 27 | 4724630 | 55.18 | 376685 |
| D1933 | SAMN34137634 | PRJNA954276 | 0.989 | Enterobacter hormaechei | - | 58 | 16 | 4651011 | 55.78 | 577620 |
| D1934 | SAMN34137633 | PRJNA954276 | 0.961 | Enterobacter hormaechei | 158 | 52 | 32 | 4761025 | 55.11 | 283527 |
| D1936 | SAMN34137629 | PRJNA954276 | 0.962 | Enterobacter hormaechei | 78 | 38 | 31 | 4723592 | 55.18 | 459610 |
| KE10068 | SAMN34137642 | PRJNA954276 | 0.989 | Enterobacter hormaechei | 141 | 54 | 49 | 4987072 | 55.34 | 301587 |
| KE10136 | SAMN34137648 | PRJNA954276 | 0.987 | Enterobacter hormaechei | 50 | 48 | 47 | 4693692 | 55.43 | 211348 |
| KE10192 | SAMN34137620 | PRJNA954276 | 0.960 | Enterobacter hormaechei | - | 45 | 56 | 4760764 | 55.29 | 194269 |
| KE10242 | SAMN34137645 | PRJNA954276 | 0.961 | Enterobacter hormaechei | 419 | 55 | 26 | 4588240 | 55.24 | 300672 |
| KE10243 | SAMN34137638 | PRJNA954276 | 0.961 | Enterobacter hormaechei | 286 | 60 | 64 | 4939572 | 55.11 | 252986 |
| KE10270 | SAMN34137619 | PRJNA954276 | 0.961 | Enterobacter hormaechei | 419 | 71 | 23 | 4587328 | 55.24 | 392533 |
| KE6463 | SAMN34137640 | PRJNA954276 | 0.961 | Enterobacter hormaechei | 118 | 39 | 60 | 4745538 | 55.04 | 188619 |
| KE6464 | SAMN34137614 | PRJNA954276 | 0.961 | Enterobacter hormaechei | 419 | 46 | 33 | 4644882 | 55.19 | 251413 |
| KE6466 | SAMN34137643 | PRJNA954276 | 0.961 | Enterobacter hormaechei | 104 | 53 | 50 | 4697156 | 55.07 | 330670 |
| KE8034 | SAMN34137632 | PRJNA954276 | 0.973 | Enterobacter hormaechei | - | 44 | 17 | 4515003 | 55.54 | 701350 |
| KE8035 | SAMN34137630 | PRJNA954276 | 0.961 | Enterobacter hormaechei | 158 | 53 | 36 | 4599220 | 55.19 | 283527 |
| KE8037 | SAMN34137631 | PRJNA954276 | 0.962 | Enterobacter hormaechei | 78 | 56 | 29 | 4725260 | 55.16 | 376685 |
| KE8125 | SAMN34137613 | PRJNA954276 | 0.987 | Enterobacter hormaechei | 742 | 71 | 24 | 4785289 | 55.83 | 610143 |
| KE8394 | SAMN34137605 | PRJNA954276 | 0.962 | Enterobacter hormaechei | 126 | 45 | 25 | 4577861 | 55.34 | 440683 |
| KE8451 | SAMN34137616 | PRJNA954276 | 0.987 | Enterobacter hormaechei | 106 | 45 | 34 | 4833080 | 55.49 | 431296 |
| KE8544 | SAMN34137627 | PRJNA954276 | 0.988 | Enterobacter hormaechei | 50 | 35 | 39 | 4893305 | 55.35 | 310917 |
| KE9056 | SAMN34137604 | PRJNA954276 | 0.961 | Enterobacter hormaechei | 135 | 53 | 49 | 4907435 | 55.15 | 281233 |
| KE9297 | SAMN34137625 | PRJNA954276 | 0.962 | Enterobacter hormaechei | - | 30 | 39 | 4904338 | 55.15 | 281431 |
| KE9302 | SAMN34137641 | PRJNA954276 | 0.974 | Enterobacter hormaechei | 295 | 31 | 24 | 4620349 | 55.5 | 444921 |
| KE9311 | SAMN34137636 | PRJNA954276 | 0.974 | Enterobacter hormaechei | 295 | 47 | 22 | 4620282 | 55.49 | 474098 |
| KE9315 | SAMN34137622 | PRJNA954276 | 0.962 | Enterobacter hormaechei | 78 | 36 | 35 | 4761472 | 55.15 | 274364 |
| KE9318 | SAMN34137611 | PRJNA954276 | 0.962 | Enterobacter hormaechei | 78 | 59 | 33 | 4761875 | 55.15 | 274364 |
| KE9319 | SAMN34137602 | PRJNA954276 | 0.962 | Enterobacter hormaechei | 78 | 61 | 33 | 4761633 | 55.15 | 274364 |
| KE9320 | SAMN34137617 | PRJNA954276 | 0.962 | Enterobacter hormaechei | 78 | 50 | 34 | 4761397 | 55.15 | 376685 |
| KE9322 | SAMN34137649 | PRJNA954276 | 0.962 | Enterobacter hormaechei | 78 | 48 | 34 | 4761629 | 55.15 | 376697 |
| KE9348 | SAMN34137623 | PRJNA954276 | 0.962 | Enterobacter hormaechei | 78 | 53 | 33 | 4740024 | 55.17 | 376685 |
| KE9404 | SAMN34137603 | PRJNA954276 | 0.962 | Enterobacter hormaechei | 78 | 53 | 28 | 4745289 | 55.18 | 356421 |
| KE9432 | SAMN34137644 | PRJNA954276 | 0.962 | Enterobacter hormaechei | 78 | 47 | 31 | 4762972 | 55.16 | 338858 |
| KE9436 | SAMN34137615 | PRJNA954276 | 0.961 | Enterobacter hormaechei | 145 | 63 | 53 | 4863429 | 54.92 | 209218 |
| KE9453 | SAMN34137639 | PRJNA954276 | 0.962 | Enterobacter hormaechei | 78 | 66 | 30 | 4762932 | 55.15 | 376685 |
| KE9477 | SAMN34137608 | PRJNA954276 | 0.962 | Enterobacter hormaechei | 78 | 66 | 28 | 4764516 | 55.15 | 482741 |
| KE9480 | SAMN34137650 | PRJNA954276 | 0.962 | Enterobacter hormaechei | 102 | 67 | 30 | 4595574 | 55.21 | 333578 |
| KE9483 | SAMN34137601 | PRJNA954276 | 0.988 | Enterobacter hormaechei | 50 | 49 | 30 | 4829036 | 55.36 | 409484 |
| KE9580 | SAMN34137624 | PRJNA954276 | 0.989 | Enterobacter hormaechei | 350 | 47 | 54 | 5061574 | 55.37 | 366757 |
| KE9607 | SAMN34137647 | PRJNA954276 | 0.991 | Enterobacter hormaechei | 45 | 49 | 39 | 4868614 | 55.44 | 385950 |
| KE9608 | SAMN34137621 | PRJNA954276 | 0.960 | Enterobacter hormaechei | 97 | 64 | 54 | 4769822 | 54.98 | 147102 |
| KE9632 | SAMN34137606 | PRJNA954276 | 0.972 | Enterobacter hormaechei | 66 | 39 | 38 | 4879175 | 55.26 | 486681 |
| KE9726 | SAMN34137635 | PRJNA954276 | 0.987 | Enterobacter hormaechei | 742 | 39 | 28 | 4787642 | 55.54 | 371900 |
| KE9770 | SAMN34137618 | PRJNA954276 | 0.962 | Enterobacter hormaechei | 78 | 75 | 27 | 4732241 | 55.24 | 391159 |
| KE9819 | SAMN34137637 | PRJNA954276 | 0.978 | Enterobacter hormaechei | 68 | 49 | 37 | 4811042 | 55.35 | 299720 |
| KE9859 | SAMN34137600 | PRJNA954276 | 0.988 | Enterobacter hormaechei | 133 | 45 | 43 | 4923073 | 55.34 | 413128 |
| KE9967 | SAMN34137612 | PRJNA954276 | 0.989 | Enterobacter hormaechei | 776 | 38 | 20 | 4737865 | 55.49 | 565527 |
| D1853 | SAMN34137651 | PRJNA954276 | 0.997 | Enterobacter kobei | 32 | 176 | 37 | 4999295 | 54.86 | 374311 |
| KE9561 | SAMN34137652 | PRJNA954276 | 0.990 | Enterobacter kobei | 56 | 43 | 53 | 4863735 | 54.84 | 194840 |
| KE10063 | SAMN34137657 | PRJNA954276 | 0.996 | Escherichia coli | 10 | 59 | 122 | 5086632 | 50.77 | 119182 |
| KE10149 | SAMN34137653 | PRJNA954276 | 0.976 | Escherichia coli | 62 | 40 | 169 | 5266416 | 50.53 | 86367 |
| KE8482 | SAMN34137658 | PRJNA954276 | 0.973 | Escherichia coli | 131 | 79 | 93 | 5090119 | 50.73 | 222568 |
| KE8965 | SAMN34137656 | PRJNA954276 | 0.978 | Escherichia coli | 69 | 43 | 45 | 4976297 | 50.64 | 314491 |
| KE9057 | SAMN34137659 | PRJNA954276 | 0.978 | Escherichia coli | 69 | 59 | 42 | 4975048 | 50.64 | 308316 |
| KE9838 | SAMN34137654 | PRJNA954276 | 0.997 | Escherichia coli | 10 | 44 | 127 | 5225383 | 50.67 | 112976 |
| KE9968 | SAMN34137655 | PRJNA954276 | 0.968 | Escherichia coli | 3910 | 42 | 67 | 5078821 | 50.29 | 406135 |
| KE10114 | SAMN34137662 | PRJNA954276 | 0.985 | Klebsiella aerogenes | 593 | 38 | 23 | 5366298 | 54.82 | 1065943 |
| KE9544 | SAMN34137666 | PRJNA954276 | 0.985 | Klebsiella aerogenes | 93 | 63 | 24 | 5260600 | 55.11 | 399635 |
| KE9586 | SAMN34137665 | PRJNA954276 | 0.985 | Klebsiella aerogenes | 93 | 40 | 23 | 5261530 | 55.11 | 473119 |
| KE9596 | SAMN34137664 | PRJNA954276 | 0.973 | Klebsiella aerogenes | 177-2LV | 38 | 21 | 5188608 | 54.87 | 551066 |
| KE9791 | SAMN34137667 | PRJNA954276 | 0.985 | Klebsiella aerogenes | 205 | 79 | 34 | 5174753 | 54.96 | 296554 |
| KE9793 | SAMN34137668 | PRJNA954276 | 0.985 | Klebsiella aerogenes | 205 | 60 | 36 | 5174344 | 54.96 | 296554 |
| KE9813 | SAMN34137663 | PRJNA954276 | 0.985 | Klebsiella aerogenes | 205 | 41 | 36 | 5174728 | 54.96 | 296554 |
| KE9820 | SAMN34137661 | PRJNA954276 | 0.985 | Klebsiella aerogenes | 205-1LV | 35 | 40 | 5177885 | 54.96 | 379331 |
| KE9860 | SAMN34137660 | PRJNA954276 | 0.985 | Klebsiella aerogenes | 205 | 44 | 38 | 5174816 | 54.96 | 336598 |
| KE10118 | SAMN34137670 | PRJNA954276 | 0.964 | Serratia nevei | - | 38 | 22 | 5204121 | 59.99 | 708163 |
| KE10127 | SAMN34137669 | PRJNA954276 | 0.964 | Serratia nevei | - | 41 | 22 | 5204168 | 59.99 | 708162 |
| KE10198 | SAMN34137671 | PRJNA954276 | 0.964 | Serratia nevei | - | 71 | 21 | 5203118 | 59.99 | 708163 |
| KE7482 | SAMN34137672 | PRJNA954276 | 0.988 | Serratia ureilytica | - | 52 | 20 | 5086748 | 59.67 | 756063 |
| KE7501 | SAMN34137673 | PRJNA954276 | 0.988 | Serratia ureilytica | - | 55 | 19 | 5088547 | 59.67 | 758098 |

**Supplementary figure 1**. **Resistome of the full cohort*.*** Isolates are ordered on a phylogenetic tree built on the 76 core genes and 12455 hqSNPs. The presence of antimicrobial resistance genes is symbolized with a black square.


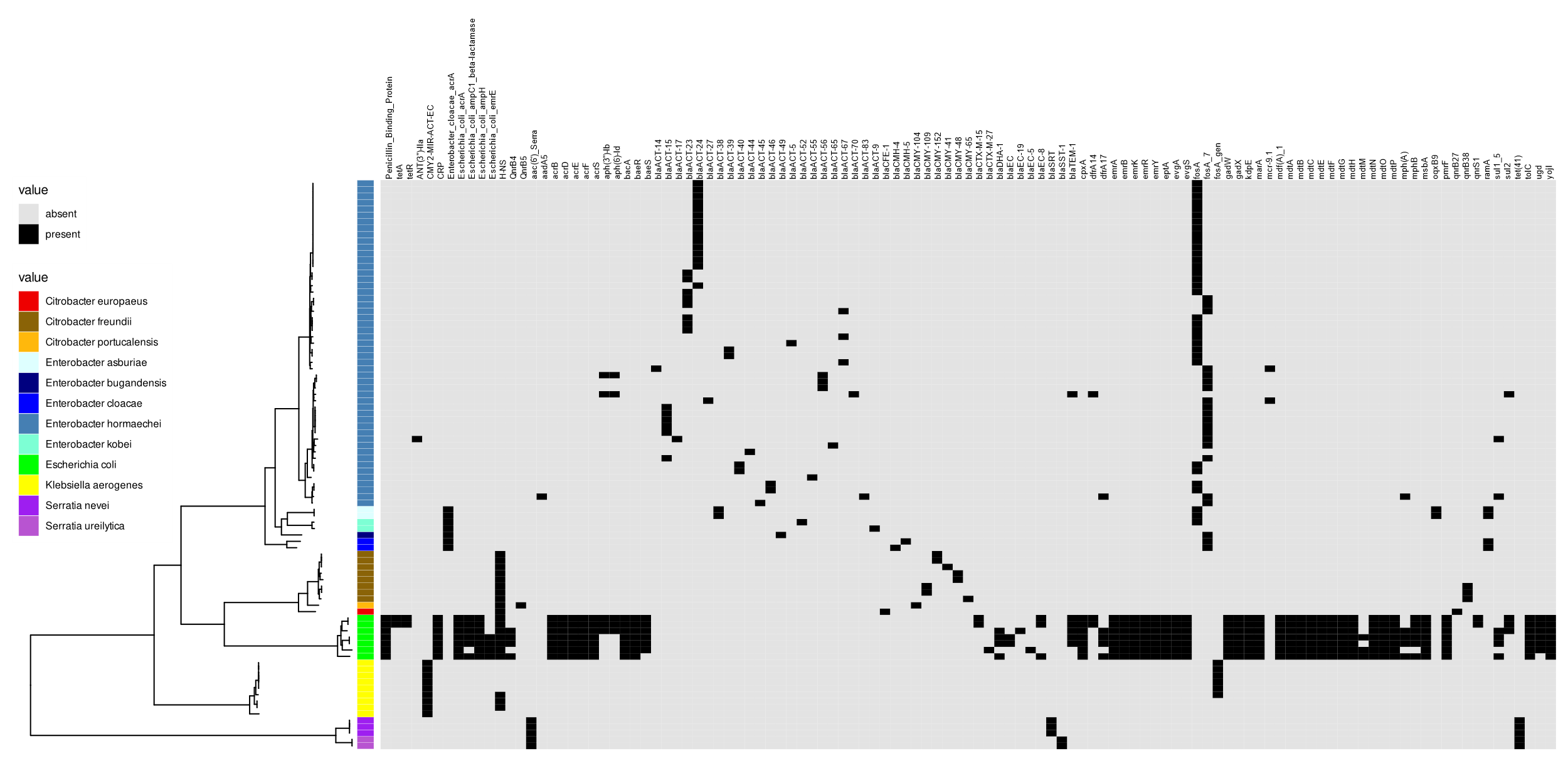

Supplement: Supplementary Appendix — Supplemental methods, Table S1, and Fig. S1. [file spectrum.02038-23-s0001.docx]
